# Optimizing Cord Clamping in Preterm Infants: One Strategy Does Not Fit All

**DOI:** 10.3389/fped.2019.00046

**Published:** 2019-02-27

**Authors:** Michael P. Meyer, Elizabeth Nevill

**Affiliations:** ^1^Middlemore Hospital, Auckland, New Zealand; ^2^Department of Paediatrics, The University of Auckland, Auckland, New Zealand

**Keywords:** preterm, delayed cord clamping (DCC), transition, resuscitation, umbilical cord milking

Delaying clamping of the umbilical cord for at least 30 s after preterm birth is currently recommended by the International Liaison Committee on Resuscitation (ILCOR) and is incorporated into many guidelines (1). A recent meta-analysis of randomized trials showed a significant reduction in neonatal mortality in preterm infants when delayed cord clamping (DCC) of > 30 s was undertaken with a number needed to benefit 33 (2). Almost 1,400 preterm infants have been allocated to receive DCC in these studies. Whilst the procedure was generally well-tolerated, some preterm infants were not vigorous and did not receive the intervention. In addition, a group of infants were deemed ineligible because of maternal conditions. For these and a variety of other reasons a significant proportion of infants in the two largest studies (Australian Placental Transfusion Study and the Cord Pilot Trial) either did not complete DCC as specified in the study protocols or were ineligible (3, 4). Even after randomization, over 20% of infants in these two studies did not get more than 30 s DCC and may not have derived benefit. There is evidence from observational studies that infants that are not vigorous and breathing during DCC are sicker and likely to have worse outcomes (5, 6). There is uncertainty over the best course of action in these infants. Should infants receive resuscitation support and remain attached to the placenta, or should the procedure be terminated, and/ or the cord milked? If the cord is to be milked, should this be done whilst it is attached to the placenta or should it be milked after clamping? Are there options to ensure placental transfusion in infants excluded for maternal reasons? Whilst many of these questions are unanswered, we review current evidence in these different situations.

Although ILCOR has regarded delayed cord clamping (DCC) as clamping after 30 s, (1), the optimal timing has not been determined. In a recent meta-analysis, outcomes of preterm infants did not show differences based on the timing of cord clamping between 30 and 120 s or more, although in most studies clamping occurred between 45 and 60 s (2). Guidelines and practices show considerable variation and it has been proposed that achieving lung expansion prior to clamping is a more important physiologic consideration (7).

DCC in term infants has been shown to have benefits in regard to prevention of anemia and iron deficiency (8). Such effects may be of greater benefit in resource-poor settings (9, 10). There have been relatively few studies of the effect of DCC in moderate to late preterm infants (over 32 weeks), although observational studies have reported a reduction in respiratory distress and need for resuscitation (11, 12).

It is preterm infants < 32 weeks that are most likely to benefit from DCC and numerous randomized studies, most of which were small in size, have been carried out. More recently, several larger studies, the Australian Placental Transfusion Study (APTS) and the Cord Pilot Trial have been performed (3, 4). In the APTS study, which randomized 1,566 infants <30 weeks to 60 s or more of DCC or Immediate Cord Clamping (ICC) ≤10 s, the primary outcome was death or major morbidity (defined as severe brain injury, necrotizing enterocolitis (NEC), severe retinopathy or late onset sepsis) at 36 weeks. There were no significant differences in mortality or other major morbidities. A systematic review, however, noted reduced hospital mortality in infants <37 weeks undergoing DCC of 30 s or more compared to ICC (2). There were 2,834 preterm infants in 18 randomized trials; DCC took place ≥45 s and up to 60 s for the majority of infants. Hospital mortality was reduced in the group undergoing DCC (RR 0.69, 95% CI 0.52 to 0.91) compared to clamping before 30 s. There was no heterogeneity (*I*^2^ = 0) and the GRADE quality of evidence was high. In the subgroup of infants ≤28 weeks gestation, DCC reduced hospital mortality (RR 0.70, 95% CI 0.51 to 0.95). Apart from reduced receipt of blood transfusion, other neonatal outcomes were not improved. An increase in cases of polycythaemia and a small increase in serum bilirubin levels were noted but no unwanted clinical effects were apparent. The proportion of infants with an Apgar score <4 at 1 min was reduced (RR 0.82, 95% CI 0.67 to 1.00, *P* = 0.05, borderline significance). There is currently little information on neurodevelopment.

## DCC in Preterm Infants: The Current Standard

As a result of the above studies, DCC is the current method of choice in preterm infants. The strength of evidence means that future studies comparing DCC with ICC are unnecessary. What can be expected from getting this research into practice?

## How Often do Preterm Infants Receive the Intervention in Practice?

This can be divided into experience from randomized trials or observational studies.

### Randomized Trials Where DCC Was an Intended Intervention

In the APTS, of 784 infants <30 weeks randomized to DCC, the planned intervention of 60 s DCC was not achieved in all infants and 163 (21%) had up to 30 s DCC (3). This does not include those that were excluded because of maternal reasons (unspecified).

In the Cord Pilot Trial, exclusions for maternal reasons (prior to randomization) were not detailed, but of 945 women approached 41 (4.3%) either were not eligible or were excluded for clinical reasons (4). Altogether, 276 preterm infants <32 weeks were randomized with 135 allocated to receive >120 s DCC with immediate neonatal care to be given with the cord intact. Of the 135, 31 (23%) did not receive longer than 30 s DCC (12.6 % were clamped before 10 s). Of those where DCC was curtailed, 13/135 (10%) were either born with intact membranes and the placenta or there was an abruption.

There were 2 other multicentre randomized trials with over 150 infants. In one study, eligible infants <32 weeks were randomized to DCC for 60 s or positive pressure T piece ventilation for 60 s prior to clamping (V-DCC), 24% were excluded prior to randomization for placental or maternal reasons, although all randomized infants received the intervention (13). In the other study, DCC for 60 s was compared with cord milking. Prior to randomization 15% were excluded and a further 17% did not receive the planned 60 s DCC. By comparison, in only 2% of cases was milking not achieved (14).

### Observational and Implementation Studies Where DCC Was an Intended Intervention

Liu et al. compared a 2 year period before and after implementation of a protocol for 30 s DCC in infants 23–32 weeks gestation (15). Compliance improved over time. Exclusion criteria included multiple birth, general anesthesia, major fetal anomalies, or no intent to resuscitate (numbers not supplied). The procedure could be terminated at any time at the discretion of attending clinicians. In 100 of 187 (53%) eligible infants, 30 s DCC was achieved.

Aziz et al. implemented a policy of 45 s DCC in preterm infants 23–33 weeks gestation (16). There were 480 eligible because of gestation. Following exclusion of 131/480 (27%) for obstetric reasons, only 349 were deemed eligible for DCC. However, clinicians attempted DCC in 236 infants and in 12 cases (5%), the procedure was abandoned prior to 45 s. Therefore, of the initial 480 infants, only 224 (46.6%) achieved 45 s DCC.

In the study by Chiruvolu et al. of 96 infants <32 weeks, 36 (34%) were deemed ineligible, mainly for maternal indications (17).

In summary, in the above studies, percentages of infants deemed ineligible for maternal reasons varied from 4 to 34% with a mean of 19% (Table 1). In addition, while the percentages of eligible infants that actually achieved DCC were variable, in the largest randomized trials, over 20% did not achieve more than 30 s DCC. Currently we estimate that 19% (exclusion for maternal reasons) plus 20% where DCC was <30 s or 39% of infants <32 weeks did not get more than 30 s DCC. However, in several studies compliance increased over time (15, 16, 18).

**Table 1:** Summary data from large studies detailing implementation of delayed cord clamping.

**Study**	**Type**	**Sample size**	**Intervention allocation (number per group)**	**Excluded for maternal or placental reason prior to study**	**Number (%) did not receive intended intervention**	**Number (%) did not receive control intervention**	**Notes**
APTS 2017 <30 weeks	RCT	1566	DCC ≥ 60 s vs. ICC (784/ 782)	Not specified	163/784 (21% ≤30 sec; 26% ≤60 s DCC)	40/782 (5%) (ICC)	21% received ≤ 30 s DCC, due to clinical concerns
Cord pilot trial 2018 <32 weeks	RCT	276	DCC ≥ 2 min vs. ICC (137/139)	41/945 (4.3%)	30/135 (23%) <30 s (DCC)	15/139 (11%) (ICC)	55 < 2 min DCC
Katheria 2016 <32 weeks	RCT	150	V-DCC vs. DCC 60 s (75/75)	55/230 (24%)	0/75 (0%) (V-DCC)	0/75 (0%) (DCC)	Placental/ maternal exclusions for twins, PPROM, imminent birth
Katheria 2015 <32 weeks	RCT	197	UCM vs. DCC ≥45 s (98/ 99)	53/342 (15%)	2/98 (2%) (milking)	17/99 (17%) (DCC)	Monochorionic twins, imminent birth, placental reasons
Liu 2017 <32 weeks	OBS	427	DCC 30 s vs. ICC (187/240)	Not specified	87/187 (47%) (DCC)	0/240 (0%) (ICC)	Protocol compliance improved over time
Aziz 2012 <33 weeks	IMP	480	DCC 45 s vs. ICC (236/113)	131/480 (27%)	125/349 (36%)	0/113 (0%)	DCC initiated in 236 cases and abandoned in 5% by 20 sec
Chiruvolu 2015 <32 weeks	IMP	148	DCC 45 s vs. ICC (60/88)	33/96 (34%)	0%	0%	36/96 ineligible mainly for maternal reasons. Multiple births excluded

*APTS, Australian Placental Transfusion Study; RCT, randomized controlled trial; OBS, observational study; IMP, implementation study; DCC, delayed cord clamping; ICC, immediate cord clamping; V-DCC, respiratory support during DCC; UCM, umbilical cord milking; PPROM, preterm prolonged rupture of membranes*.

## Is it Possible to Predict in Which Preterm Infants DCC will be Curtailed?

Most preterm infants quickly establish respiration (13, 16), but there is a group that do not. In the APTS, early cessation of DCC was more likely in infants <27 weeks, with lower birth weight and in multiple pregnancy (3). Observational studies have confirmed that failure to establish respiration during DCC was linked to worse outcomes (5, 6).

## Directions of Current Research

Studies have embarked on different pathways to overcome the problem of perceived ineligibility for DCC. One approach is to undertake initial resuscitation with the cord intact and prior to delivery of the placenta. Another broad strategy is that of milking the cord either before or after it is cut. Removal of the baby and placenta together and resuscitation with the placenta attached is also being trialed (19).

## Provision of Respiratory Support Before Cord Clamping in Preterm Infants

This has been the subject of a recent Cochrane review (20) and there was one randomized trial in infants <32 weeks comparing 60 s DCC with DCC with CPAP or positive pressure ventilation (13). Outcomes were not significantly different. In this study, 90% of the infants were breathing prior to cord clamping and this has been confirmed in several observational studies as noted previously. In the Cord Pilot Trial, immediate cord clamping (ICC) was compared with DCC in infants <32 weeks (4). It was planned to undertake initial resuscitation (including intubation and surfactant administration if needed) whilst DCC was taking place. The procedure was found to be feasible and safe. It should also be noted that many apnoeic infants undergoing DCC will respond to tactile stimulation and may not require more intensive resuscitation to establish respiration (13).

The concept of physiologic based cord clamping relates to clamping the cord only after respiration is established, rather than waiting for a specific time. There is resurgence of interest following animal experiments and the concept is to minimize changes in cardiac output and cerebral blood flow. The placenta remains part of the transitional circulation while lung expansion occurs (21). Preliminary studies in preterm infants have described the technique, which is feasible, and there is ongoing research in progress (22–24). In a study where clamping was performed after gas exchange was established, it was noted that bradycardia was less common in the first minute after birth than after ICC in historical controls (25). Providing this “placental support” before clamping the cord has promise in facilitating a smoother transition and preventing fluctuations in cerebral blood flow.

## Cord Milking as an Alternative to DCC in Preterm Infants

Umbilical cord milking (UCM) refers to holding the umbilical cord close to the placenta and squeezing the blood toward the infant while the cord remains attached to the placenta. The cord is milked 3 to 4 times, allowing it to refill for 2–5 s in between. Meta -analysis of milking compared to DCC indicated there may be some advantages (26). In two randomized controlled trials, 255 infants preterm infants <33 weeks were studied. The cord was milked as described and DCC was 45 s in 1 study and 30 s in the other. In the milking group there was a reduction in intraventricular hemorrhage (IVH) of any grade (RR 0.45 0.21–0.98) and a significantly higher proportion of infants had a Bayley III score of 85 or more for cognition and language at 2 yrs. The quality of evidence was regarded as low because of small numbers and wide confidence intervals. Of potentially eligible pregnancies, 15% were excluded for maternal reasons. More infants underwent cord milking and only 2/98 did not receive the intervention compared to 17/99 in the DCC group (*p* < 0.001). Before more widespread introduction, however, results from animal experiments need to be considered. Cord milking in a preterm lamb model (27) was associated with fluctuations in carotid artery pressure and flow and if the same occurs in preterm infants, this could predispose to cerebral injury. Nevertheless, results of clinical trials in preterm infants to date have not demonstrated harm, and, compared to DCC, there was higher systemic blood flow and blood pressure (14).

## Cord Milking after Clamping in Preterm Infants

The umbilical cord can be milked after clamping by leaving a longer segment (20 cm or more), unraveling the cord and then milking 2–3 times toward the infant (28). In a novel study, infants <30 weeks had milking of the cut umbilical cord performed if they were not spontaneously breathing and were deemed in need of resuscitation (29). Maternal complications were not an exclusion criterion. There were 158 infants eligible for cord milking and 160 controls. The composite outcome (death, severe IVH, NEC) was significantly higher in the controls (39% vs. 22%; OR1.81 95% CI 1.06–3.10). For infants <27 weeks the composite outcome was significantly worse in the control group (*p* < 0.05). No adverse events were reported and there was no difference in neonatal resuscitation or Apgar scores.

A recent study examined the effects of milking the cut cord in preterm infants in need of resuscitation (did not breathe or cry at birth), and found no adverse effects and significantly higher hemoglobin and serum ferritin levels in the milked group at 6 weeks postnatal age (30).

## Current state in preterm infants

Even though a significant percentage of infants did not receive more than 30 s DCC in the 2 largest randomized trials, meta-analysis based on intention to treat, demonstrated reduced mortality. This suggests that the DCC effect size on mortality is quite large but it remains to be seen what should be done for the high percentage of infants where DCC was not carried out. Providing respiratory support prior to clamping will likely reduce this number and early indications are that DCC with support may be achievable in most cases. Whilst it seems that individual babies may benefit, evidence from randomized trials is awaited. Limited data indicates UCM is more likely to be achieved than DCC. However, before UCM could be the method of choice, larger trials will be needed to determine that UCM is not inferior to DCC.

Where infants are not vigorous by 20–30 s of DCC, cord milking may be a reasonable alternative but is not currently recommended for infants <29 weeks gestation (1). For situations where infants are deemed ineligible for DCC (mainly for maternal reasons), milking could be carried out and this forms part of the Italian recommendations on cord management (31). Where conditions preclude uncut cord milking, there is limited evidence that milking the cut cord may still be preferable to ICC with no milking.

It is apparent that one size does not fit all with regard to strategies to ensure optimal timing of cord clamping in the different situations accompanying preterm birth.

## Author Contributions

MM and EN conceived the article. MM wrote the first draft. EN prepared the Table. Both authors critically edited the article.

### Conflict of Interest Statement

The authors declare that the research was conducted in the absence of any commercial or financial relationships that could be construed as a potential conflict of interest.

## References

[1] Perlman JM Wyllie J Kattwinkel J Wyckoff MH Aziz K Guinsburg R. Part 7: neonatal resuscitation: 2015 international consensus on cardiopulmonary resuscitation and emergency cardiovascular care science with treatment recommendations. Circulation (2015) 132:S204–41. 10.1161/CIR.0000000000000276 26472855

[2] Fogarty M Osborn DA Askie L Lene Seider A Hunter K Lui K. Delayed vs. early umbilical cord clamping for preterm infants: a systematic review and meta-analysis. Am J Obstet Gynecol. (2018) 218:1–18. 10.1016/j.ajog.2017.10.231 29097178

[3] Tarnow-Mordi W Morris J Kirby A Robledo K Askie L Brown R. Delayed versus immediate cord clamping in preterm infants. N Engl J Med. (2017) 377:2445–55. 10.1056/NEJMoa1711281 29081267

[4] Duley L Dorling J Pushpa-Rajah A Oddie SJ Yoxall CW Shoemaker B. on behalf of the Cord Pilot Trial Collaborative Group. Randomised trial of cord clamping and initial stabilisation at very preterm birth. Arch Dis Child Fetal Neonatal Ed. (2018) 103:F6–14. 10.1136/archdischild-2016-312567 28923985 PMC5750367

[5] Nevill E Meyer MP. Effect of delayed cord clamping on breathing and transition at birth in very preterm infants. Early Hum Dev. (2015) 91:407–11. 10.1016/j.earlhumdev.2015.04.013 25984654

[6] Ersdal HL Linde J Mduma E Auestad B Perlman J. Neonatal outcome following cord clamping after onset of spontaneous respiration. Pediatrics (2014) 134: 265–72. 10.1542/peds.2014-0467 25022738

[7] Hooper SB Polglase GR te Pas AB. A physiological approach to the timing of umbilical cord clamping at birth. Arch Dis Child Fetal Neonatal Ed. (2015)100:F355–60. 10.1136/archdischild-2013-305703 25540147

[8] Ashish KC Rana N Målqvist M Ranneberg LJ Subedi K Andersson O. Effects of delayed umbilical cord clamping vs. early clamping on anemia in infants at 8 and 12 months: a randomized clinical trial. JAMA Pediatr. (2017) 171:264–70. 10.1001/jamapediatrics.2016.3971 28114607

[9] Van Rheenen PF Brabin BJ. A practical approach to timing cord clamping in resource poor settings. BMJ (2006) 333:954–8. 10.1136/bmj.39002.389236.BE 17082547 PMC1633763

[10] Andersson O Domellöf M Andersson D Hellström-Westas L. Effect of delayed vs. early cord clamping on iron status and neurodevelopment at age 12 months. A randomized controlled trial. JAMA Pediatr. (2014) 168:547–54. 10.1001/jamapediatrics.2013.4639 24756128

[11] Chiruvolu A Qin H Nguyen ET Inzer RW. The effect of delayed cord clamping on moderate and early late-preterm infants. Am J Perinatol. (2018) 35:286–91. 10.1055/s-0037-1607222 28958092

[12] Kaempf JW Tomlinson MW Kaempf AJ Wu Y Wang L Tipping N. Delayed umbilical cord clamping in premature neonates. Obstet Gynecol. (2012) 120:325–30. 10.1097/AOG.0b013e31825f269f 22825092

[13] Katheria A Poeltler D Durham J Steen J Rich W Arnell K. Neonatal resuscitation with an intact cord: a randomized clinical trial. J Pediatr. (2016) 178:75–80. e3. 10.1016/j.jpeds.2016.07.053 27574999 PMC5527831

[14] Katheria AC Truong G Cousins L Oshiro B Finer NN. Umbilical cord milking versus delayed cord clamping in preterm infants. Pediatrics (2015) 136:61–9. 10.1542/peds.2015-0368 26122803 PMC4485011

[15] Liu LY Feinglass JM Khan JY Gerber SE Grobman Yee LM. Evaluation of introduction of delayed cord clamping protocol for premature neonates in a high-volume maternity centre. Obstet Gynaecol. (2017) 129:835–43. 10.1097/AOG.0000000000001987 28383377 PMC5400681

[16] Aziz K Chinnery K Lacaze-Masmonteil T. A single-centre experience of implementing delayed cord clamping in babies born at less than 33 weeks gestational age. Adv Neonatal Care (2012) 12:371–6. 10.1097/ANC.0b013e3182761246 23187645

[17] Chiruvolu A Tolia VN Qin H Leal Stone G Rich D Conant RJ. Effect of delayed cord clamping on very preterm infants. Am J Obstet Gynecol. (2015) 213:676.e1–7. 10.1016/j.ajog.2015.07.016 26196456

[18] Balakrishnan M Falk-Smith N Detman LA Miladinovic B Sappenfield WM Curran JS. Promoting teamwork may improve infant care processes during delivery room management: florida perinatal quality collaborative approach. J Perinatol. (2017) 37:886–92. 10.1038/jp.2017.27 28406486

[19] Kuehne B Kirchgaessner C Becker I Kuckelkorn M Valter M Kribs A Mask continuous positive airway pressure therapy with simultaneous extrauterine placental transfusion for resuscitation of preterm infants - a preliminary study. Biomed Hub. (2018) 3:1–1. 10.1159/000488926 PMC6945906 31988958

[20] Meyer MP Nevill E Wong MM. Provision of respiratory support compared to no respiratory support before cord clamping for preterm infants. Cochrane Database Syst Rev. (2018) 3:CD012491. 10.1002/14651858.CD012491.pub2 29516473 PMC6494179

[21] Bhatt S Alison BA Wallace EM Crossley KJ Gill AW Kluckow M. Delaying cord clamping until ventilation onset improves cardiovascular function at birth in preterm lambs. J Physiol. (2013) 591. 2113–26. 10.1113/jphysiol.2012.250084 23401615 PMC3634523

[22] Brouwer E Knol R Vernooij ASN van den Akker T Vlasman PE Klumper FJCM. Physiological-based cord clamping in preterm infants using a new purpose-built resuscitation table: a feasibility study. Arch Dis Child Fetal Neonatal Ed. (2018). 10.1136/archdischild-2018-315483. [Epub ahead of print]. 30282674 PMC6764254

[23] Knol R Brouwer E Vernooij ASN Klumper FJCM DeKoninck P Hooper SB. Clinical aspects of incorporating cord clamping into stabilisation of preterm infants. Arch Dis Child Fetal Neonatal Ed. (2018) 103:F493–7. 10.1136/archdischild-2018-314947 29680790 PMC6109247

[24] Pratesi S Montano S Ghirardello S Mosca F Boni L Tofani L. Placental Circulation Intact Trial (PCI-T)-resuscitation with the placental circulation intact vs. cord milking for very preterm infants: a feasibility study. Front Pediatr. (2018) 6:364. 10.3389/fped.2018.00364 30538975 PMC6277460

[25] Blank DA Badurdeen S Kamlin OF Jacobs SE Thio M Dawson JA. Baby-directed umbilical cord clamping: a feasibility study. Resuscitation (2018) 131:1–7. 10.1016/j.resuscitation.2018.07.020 30036590

[26] Nagano N Saito M Sugiura T Miyahara F Namba F Ota E. Benefits of umbilical cord milking versus delayed cord clamping on neonatal outcomes in preterm infants: a systematic review and meta-analysis. PLoS ONE (2018) 13:e0201528. 10.1371/journal.pone.0201528 30161139 PMC6116944

[27] Blank DA Polglase GR Kluckow M Gill AW Crossley KJ Moxham A. Haemodynamic effects of umbilical cord milking in premature sheep during the neonatal transition. Arch Dis Child Fetal Neonatal Ed. (2017) 103:F539–46. 10.1136/archdischild-2017-314005 29208663 PMC6278653

[28] Hosono S Mugishima H Fujita H Hosono A Minato M Okada T. Umbilical cord milking reduces the need for red cell transfusions and improves neonatal adaption in infants born at less than 29 weeks' gestation: a randomised controlled trial. Arch Dis Child Fetal Neonatal Ed. (2008) 93:F14–9. 10.1136/adc.2006.108902 17234653

[29] Patel A Clark EAS Rodriquez CE Metz TD Abbaszadeh M Yoder BA. Effect of umbilical cord milking on morbidity and survival in extremely low gestational age neonates. Am J Obstet Gynecol. (2014) 211:519.e1–7. 10.1016/j.ajog.2014.05.037 24881823

[30] Ram Mohan G Shashidhar A Chandrakala BS Nesargi S Suman Rao PN. Umbilical cord milking in preterm neonates requiring resuscitation: a randomized controlled trial. Resuscitation (2018) 130:88–91. 10.1016/j.resuscitation.2018.07.003 29981817

[31] Ghirardello S Di Tommaso M Fiocchi S Locatelli A Perrone B Pratesi S. (2018). Italian recommendations for placental transfusion strategies. Front Pediatr.(2018) 6:372. 10.3389/fped.2018.00372 30560107 PMC6287578

